# Maintenance of muscle myosin levels in adult *C. elegans* requires both the double bromodomain protein BET-1 and sumoylation

**DOI:** 10.1242/bio.20136007

**Published:** 2013-10-31

**Authors:** Kate Fisher, Fiona Gee, Siyao Wang, Feng Xue, Stefan Knapp, Martin Philpott, Christopher Wells, Miriam Rodriguez, L. Basten Snoek, Jan Kammenga, Gino B. Poulin

**Affiliations:** 1 Faculty of Life Sciences, Michael Smith Building, University of Manchester, Oxford Road, Manchester M13 9PT, UK; 2 Nuffield Department of Clinical Medicine, Old Road Campus Research Building, University of Oxford, Oxford OX3 7BN, UK; 3 Laboratory of Nematology, Wageningen Universiteit, Droevendaalsesteeg 1, 6708 PB, Wageningen, The Netherlands

**Keywords:** Bromodomain, Sumo, Body wall muscle, *C. elegans*

## Abstract

Attenuation of RAS-mediated signalling is a conserved process essential to control cell proliferation, differentiation, and apoptosis. Cooperative interactions between histone modifications such as acetylation, methylation and sumoylation are crucial for proper attenuation in *C. elegans*, implying that the proteins recognising these histone modifications could also play an important role in attenuation of RAS-mediated signalling. We sought to systematically identify these proteins and found BET-1. BET-1 is a conserved double bromodomain protein that recognises acetyl-lysines on histone tails and maintains the stable fate of various lineages. Unexpectedly, adults lacking both BET-1 and SUMO-1 are depleted of muscle myosin, an essential component of myofibrils. We also show that this muscle myosin depletion does not occur in all animals at a specific time, but rather that the penetrance of the phenotype increases with age. To gain mechanistic insights into this process, we sought to delay the occurrence of the muscle myosin depletion phenotype and found that it requires caspase activity and MEK-dependent signalling. We also performed transcription profiling on these mutants and found an up-regulation of the FGF receptor, *egl-15*, a tyrosine kinase receptor acting upstream of MEK. Consistent with a MEK requirement, we could delay the muscle phenotype by systemic or hypodermal knock down of *egl-15*. Thus, this work uncovered a caspase- and MEK-dependent mechanism that acts specifically on ageing adults to maintain the appropriate net level of muscle myosin.

## Introduction

Controlling RAS-mediated signalling is crucial to promote or inhibit cell growth, differentiation, and apoptosis in vertebrates. Loss of control at the level of attenuation can lead to hyperactivation of the pathway and in the worst cases tumourigenesis. Over two decades of studies on RAS-mediated signalling in *C. elegans* have shown that the epigenetic landscape can impact on attenuation of the LET-60 (RAS) signalling pathway and cell fate (Andersen and Horvitz, 2007; Ceol and Horvitz, 2001; Fay and Yochem, 2007; Fisher et al., 2010; Lipsick, 2004; Lu and Horvitz, 1998; Poulin et al., 2005; Solari and Ahringer, 2000). Many of the chromatin complexes depositing or removing histone modifications have since been shown to act redundantly to prevent ectopic expression of LIN-3 (EGF) (Andersen and Horvitz, 2007; Andersen et al., 2008; Cui et al., 2006). Ectopic expression of LIN-3 can lead to over activation of the receptor tyrosine kinase, LET-23 (EGFR), and its conserved downstream cascade: LET-60/LIN-45/MEK-2/MPK-1, RAS/RAF/MEK/MAPK in mammals (Sundaram, 2006). It has also been shown that the sumoylation pathway genetically interacts with many of these chromatin complexes to attenuate LET-60 (RAS)-mediated signalling (Leight et al., 2005; Poulin et al., 2005). SUMO is a conserved short polypeptide transferred onto specific substrates (Gareau and Lima, 2010; Johnson, 2004), which can be recognised by effector proteins through SUMO interacting motifs (SIMs) (Geiss-Friedlander and Melchior, 2007; Kerscher, 2007). These effector proteins can in turn regulate specific functions such as transcription, chromatin structure, genome integrity, and DNA repair (Cubeñas-Potts and Matunis, 2013; Geiss-Friedlander and Melchior, 2007). Collectively, these studies raised the possibility that post-translational modifications of histones, such as sumoylation, methylation, and acetylation, could form a combinatorial code recognised by specialised proteins referred to as readers of the epigenetic code, which in turn would regulate transcription of genes that prevent hyperactivation of the LET-60 signalling pathway.

We set out to identify readers that recognise chromatin modifications and genetically interact with the sumoylation pathway to prevent hyperactivation of the LET-60 signalling cascade. We used RNAi to deplete all predicted readers and identified CHD-3, HPL-2, and BET-1. CHD-3 and HPL-2 are chromodomain proteins recognising methylated histone tails and were previously shown to play a role in LET-60 attenuation (Coustham et al., 2006; Solari and Ahringer, 2000). BET-1 is a conserved double bromodomain protein of the BET family required for establishment and maintenance of stable fate in various lineages (Shibata et al., 2010). BET-1 shares homology with both human BRD2 and BRD4, and is a likely homolog of BRD4 because of a putative P-TEFb interaction motif not present in BRD2 (Bisgrove et al., 2007). BET-1, like other BET proteins, physically associates with acetyl-lysines on histone tails (Shibata et al., 2010).

Low molecular weight inhibitors such as JQ1 and I-BET151 can efficiently target acetyl-lysine binding sites of BET proteins (Dawson et al., 2011; Delmore et al., 2011; Filippakopoulos et al., 2010; Nicodeme et al., 2010; Zuber et al., 2011). In multiple myeloma, the inhibition of BRD4 leads to downregulation of the oncogenes *c-MYC* and other growth promoting and apoptotic genes (Delmore et al., 2011). This specific transcriptional regulation has recently been attributed to the effect of BRD4 on super-enhancers (Lovén et al., 2013).

Herein we performed a targeted RNAi screen and identified BET-1 as a novel SUMO interactor. Unexpectedly, we found that SMO-1 and BET-1 act together to maintain net muscle myosin levels in ageing adults. We show that muscle myosin depletion requires caspase activities and the FGF receptor/MEK signalling pathway to manifest. Interestingly, human caspases are activated under muscle catabolic conditions induced by insulin resistance (Du et al., 2004).

## Materials and Methods

### Strains and general maintenance

Strains were maintained at 20°C as described (Brenner, 1974), unless stated. For full list of strains see supplementary material Table S2. Of note, the muscle phenotype has been analysed using either *bet-1(os46)* or *bet-1(gk425)* but all presented data are with *bet-1(gk425)*.

### Identification of putative readers of chromatin marks

To identify genes for use in the targeted RNAi screen, the Pfam accession number for each domain of interest was used to filter the WormBase database (release WS190), using the WormMart data mining tool.

### RNAi experiments

RNAi screens of the ∼200 gene set were performed similarly to those described previously (Kamath et al., 2003; Poulin et al., 2005). Briefly, individual cultures were used to inoculate three wells on a six-well plate (Poulin et al., 2005), around 10 synchronized *smo-1/hT2* L3-L4 stage worms were placed in the upper well for each bacterial strain and the plates maintained at 20°C. After 48 h, 5 worms from the upper well were transferred to the lower well. The F_1_ progeny were scored for the Mvp (*m*ultiple *v*entral *p*rotrusion) phenotype. RNAi clones giving Mvp in one or more *smo-1/hT2* animals and/or two or more *smo-1/smo-1* animals were selected for further analysis. These criteria took into account the 10% background Mvp in *smo-1/smo-1* animals. RNAi clones were obtained from the Ahringer RNAi library (Kamath et al., 2003) and the Vidal RNAi library (Rual et al., 2004). All positive RNAi clones were verified by sequencing. All further RNAi experiments were performed similarly as described above.

### Immunofluorescence staining of embryos and muscle myosin

Immunofluorescence of muscle myosin on embryos or adults was performed by freeze crack method as previously described (Wang et al., 2011) with the following adaptation for staining of adults: each mother was cut open in the middle using a sharp needle. Four-day old adults (wild type or mutants) were grown from L1 on OP50, picked onto slides and freeze cracked. Slides were incubated with anti-myosin heavy chain A (5–6 (1:100) or 5–14 (1:100), Developmental Studies Hybridoma Bank, University of Iowa) overnight at 4°C in a humid chamber. Following washes slides were then incubated with DyLight 594 AffiniPure Goat Anti-Mouse IgG (H+L) (1:200) (cat.115-515-146, Jackson Immunoresearch). Pictures were taken with same settings as control wild type and each batch was systematically control with wild type.

### Fluorescence Recovery After Photobleaching (FRAP)

For FRAP studies, full length *bet-1* ORF was cloned into the pcDNA6.2/N-EmGFP-DEST vector and verified by sequencing. Full length GFP-tagged *bet-1* was reverse-transfected into U2OS cells using Fugene HD (Roche) and plated into glass bottom dishes (World Precision Instruments, USA). Bromodomain FRAP assays have been previously described (Filippakopoulos et al., 2010). Briefly, FRAP was performed 24 hours after transfection using a Zeiss LSM 710 scanhead coupled to an inverted Zeiss Axio Observer.Z1 microscope (40× oil immersion objective) and an argon-ion laser (488 nm) with PMT detection set to 500–550 nm. The HDAC inhibitor SAHA was added (5 µM, 4 hours post transfection) to increase the assay window (BET binding increased) and JQ1 (2.5 µM) was added 1 hour prior to FRAP. Approximately half of the GFP positive nucleus was selected for bleaching and a time lapse series was taken to record GFP recovery using 1% of the power used for bleaching. The image datasets and fluorescence recovery data were exported from the microscope control software (ZEN 2009) into GraphPad Prism to determine the half-time for full recovery for individual cells and averages were calculated from 10–20 cells per treatment point.

### Western blot analysis

Whole worm protein extracts at the indicated stages were prepared by harvesting synchronized worms, washing the pellet in 1× PBS and boiling in 1× sample buffer containing 100 mM DTT, and sonicating. Quantification performed using ImageJ software.

### Microarrays

Sample preparation: Whole worm extracts for microarray analysis were prepared by placing ∼50 mothers on 10 10 cm NGM plates for each strain. Gravid progeny were bleached and the eggs put on NGM plates with no food. After 24 hours the synchronised L1 were washed off and placed on plates with food. Once the F_2_ progeny had reached L4 the worms were harvested, washed once in M9 buffer and frozen at −80°C.

RNA preparation: Two replicates of *bet-1/+*, *smo-1/+* mutants, and *smo-1, bet-1/+* double mutants and the corresponding balancer strain as a control were processed for microarray analysis. Nematode pellets were incubated with 1% beta-mercaptoethanol and 800 µg/mL proteinase K at 55°C, 500 rpm shaking during 60 minutes. Total RNA was extracted from these pellets using RNeasy Micro kit (Qiagen) according to manufacturer instructions.

Microarray analysis: The extracted RNA was processed for microarray performance; the platform used for that purpose was *C. elegans* (V2) Gene Expression Microarray 4×44K (Agilent technologies), following manufacturer instructions. Raw data (supplementary material Table S3) was extracted from the scanned images by the Agilent feature extract software. Data was normalized in the statistical programming environment “R” using the LIMMA package (Smyth, 2004). For within array normalization we used the Loess method and for the between array normalization we used the quantile method. No background correction was needed. A linear model was used to determine the differently expressed genes.

### Quantitative RT-PCR

Worm pellets were prepared by harvesting synchronized L4 worms, washing the pellet in 1× PBS and freezing at −80°C. Total RNA was extracted from these pellets using TRIzol (Invitrogen) and first strand cDNA synthesis was performed using the SuperScript VILO cDNA Synthesis Kit (Invitrogen), according to the manufacturer's instructions. Quantitative RT-PCR was performed using the FastStart SYBR Green Master (ROX) mix (Roche) on a StepOnePlus Real-Time PCR System (Applied Biosystems). Two biological samples for each strain were prepared, and for every biological replicate, a triplicate of two serial dilutions was analysed. *act-1* was used as the internal reference for data normalization. *mRNA* levels were determined by comparing the unknown samples to a standard curve of known relative amounts. Primers used are listed in supplementary material Table S4.

### JQ1 and U0126 treatments of nematodes

Synchromised L1 larvae were transferred onto NGM plates prepared with the indicated concentration of JQ1 (Filippakopoulos et al., 2010) or U0126 (Morgan et al., 2010).

## Results

### BET-1 genetically interacts with SUMO

Deposition and removal of post-translational modifications (PTMs) on histone tails play an important role in transcriptional regulation, which in turn impacts on the process of attenuation of LET-60-mediated signalling in *C. elegans* (Cui et al., 2006; Lipsick, 2004; Lu and Horvitz, 1998; Poulin et al., 2005). These PTMs can act by either altering the electrostatic interactions between histones and DNA or by creating recognition sites for specialised proteins often referred to as readers of the epigenetic code (Kouzarides, 2007). To test the latter mode of action, we performed an RNAi screen targeting all known predicted readers (∼200 genes; supplementary material Table S1). Since the sumoylation pathway has been shown to genetically interact with many chromatin complexes involved in attenuation of LET-60 (Leight et al., 2005; Poulin et al., 2005), we performed the screen in a SUMO-compromised strain (*smo-1_lf_ /+*). We selected our candidates according to the observation of SUMO-associated phenotypes. The main expected phenotype being the *mu*lti*v*ulvae (Muv) phenotype (Broday et al., 2004; Leight et al., 2005; Poulin et al., 2005) and its superficial manifestation the *m*ulti*v*entral *p*rotrusion phenotype (MVP) (Fisher et al., 2010); these phenotypes indicate hyperactivation of the LET-60/LIN-45/MEK-2/MPK-1 signalling cascade. We identified three candidates: two conserved chromodomain proteins: CHD-3 (Solari and Ahringer, 2000) and HPL-2 (Coustham et al., 2006); and the double bromodomain BET-1. We focused this study on BET-1, which was previously shown to recognise acetyl-lysines on histone tails and to maintain cell fate in various lineages (Shibata et al., 2010).

### BET-1 and SUMO prevent muscle myosin depletion in adults

**Fig. 1.**
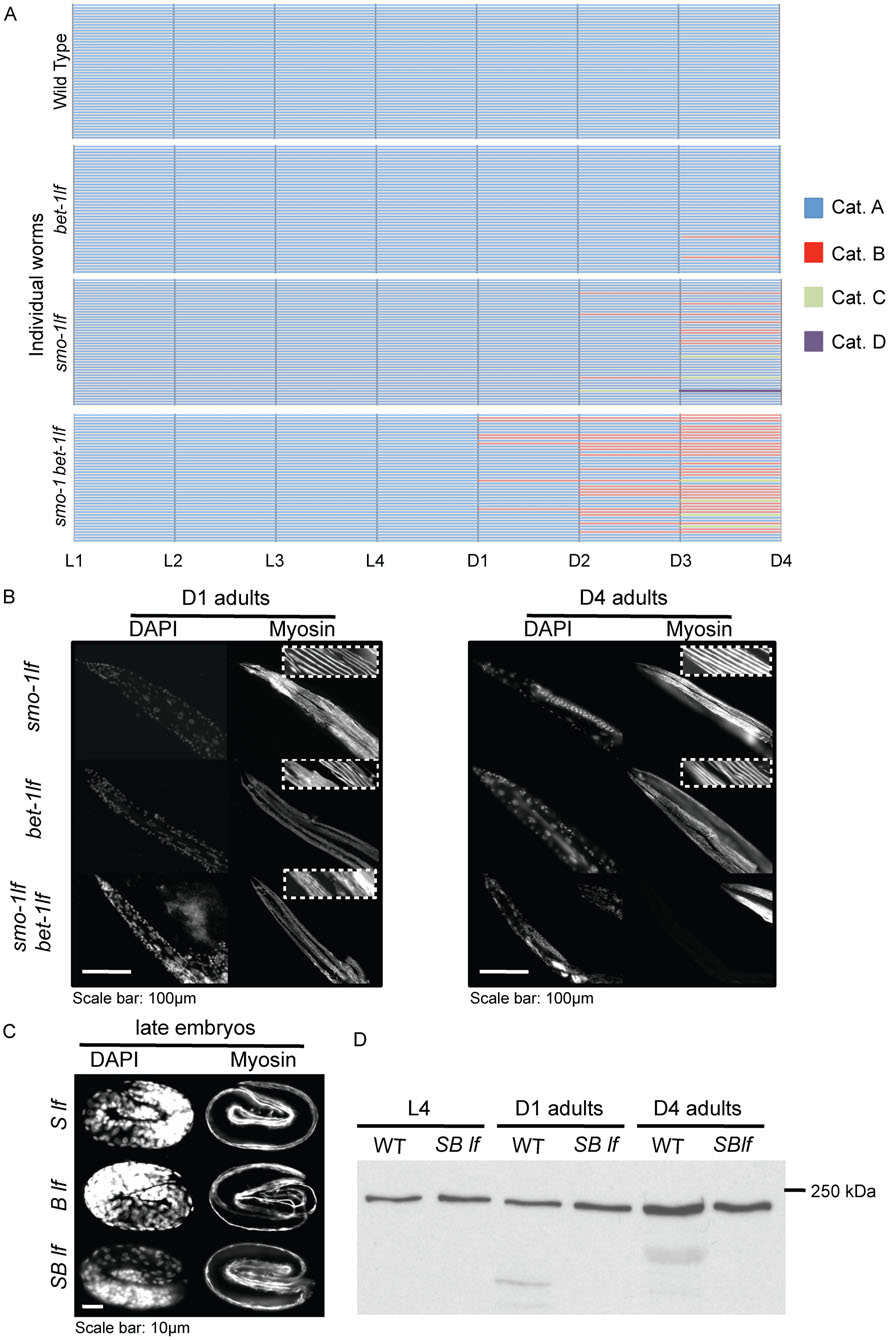
Loss of locomotion and depletion of muscle myosin in *smo-1_lf_ bet-1_lf_* animals. (A) Histograms of cloned individual animals followed using a locomotion assay from larval stage one (L1) until day four (D4) adult showing that locomotion of the double mutants is aggravated by the loss of both BET-1 and SMO-1. Blue depicts crawling animals, red, animals requiring prodding, green, immobile animals, and purple, dead animals. (B) Immunostaining against MYO-3 showing double *smo-1_lf_ bet-1_lf_* mutants displaying a high frequency of the depletion of muscle myosin phenotype at day four and five adult (see Table 1 for quantification). This is observed in all muscles but more pronounced in the posterior part of the animal. Inset is a 5.4× magnification. (C) Immunostaining against MYO-3 showing that late embryos do not display the depletion of muscle myosin phenotype. Inset is a 5.4× zoom in. (D) Western blot analysis using an anti-MYO-3 antibody showing that at day four adult, MYO-3 is depleted. Ten age-matched animals were used for each lane. We repeated the experiments four times and a representative blot is shown. Scale bars: 100 µm (B), 10 µm (C).

Following the identification of BET-1 by RNAi screening, we generated the double mutant *smo-1_lf_ bet-1_lf_* and assessed whether we could detect a genetic interaction during vulva development. Surprisingly, we could not find an interaction in the vulva. Instead, we found that the single *bet-1_lf_* mutant or RNAi against *bet-1* can produce the Muv (multiple vulvae) phenotype, but the additional loss of *smo-1* does not aggravate the Muv phenotype (data not shown). However, during these investigations, we noticed that an important proportion of these double *smo-1_lf_ bet-1_lf_* mutants lost their ability to crawl early in adulthood. We quantified this observation by assessing loss of locomotion. In this established assay (Herndon et al., 2002), locomotion can fall into three exclusive categories: fully mobile (A), mobile following prodding (B), immobile (C). This latter category is defined by animals incapable of producing a full body movement following prodding whilst remaining alive, which is determined by head movements. We measured loss of locomotion from larval stage 1 (L1) for each single mutant, the double mutant, and wild type. We found that a proportion of the single *bet-1_lf_* or *smo-1_lf_* mutant lost locomotion earlier than wild type (4.5%, *n* = 44; and 23%, *n* = 48, respectively). However, the decline in locomotion is accelerated and more penetrant in the double *smo-1_lf_ bet-1_lf_* mutants (73%, *n* = 45). The double *smo-1_lf_ bet-1_lf_* mutants are statistically different (Fisher's exact test) from N2 and the singles (*P* = 4×10^−9^ (compared with N2), 0.1×10^−9^ (compared with *bet-1_lf_*), 3×10^−6^ (compared with *smo-1_lf_*); Fig. 1A; supplementary material Fig. S1). Further, to provide an overview of the life history of each population, we present the entire dataset as box plots from day 1 till day 21. The number of days spent in each category for each strain at a specific time point can be selected (supplementary material Movie 1) or alternatively the entire 21-day assay can be viewed as a movie (supplementary material Movie 1) showing how the population for each category changes according to time. In conclusion, the locomotion assay shows that BET-1 and SMO-1 can act individually and in cooperation to prevent loss of locomotion in adults.

Time-associated loss of locomotion is a natural phenomenon attributed to a failure of maintaining a functional ageing muscle mass (Herndon et al., 2002). We therefore explored the possibility that the *bet-1_lf_* and *smo-1_lf_* mutants are experiencing a decline in muscle functionality. To test this, we directly measured muscle myosin (MYO-3), a major component of myofibrils, using immunofluorescence stainings and Western blot analysis. We first surveyed the levels of adult muscle myosin by immunostainings on wild type animals *versus* singles and double mutants. We used a modified freeze-crack method, which involves cutting open the mothers roughly in the middle, allowing the antibody to penetrate thoroughly the muscles. All wild type animals are successfully stained using this adaptation. At day one of adulthood, we found that the MYO-3 signal was depleted in 4.7% of double *smo-1_lf_ bet-1_lf_* mutants while all wild type animals and the respective single mutants all remained positive for MYO-3 (Fig. 1B). Interestingly, at day four of adulthood, the phenotype reached a penetrance of 37.5% for *smo-1_lf_ bet-1_lf_* animals. A slight increase was observed with single mutants (*bet-1_lf_* mutants: 2.7%; *smo-1_lf_* mutants: 4.8%) but no effect was found on wild type (Fig. 1B; Table 1). One day later at day five, the double *smo-1_lf_ bet-1_lf_* reached 42.5% and the respective single mutants about 10% (Fig. 1B). Depletion was never observed in late embryos (Fig. 1C). Taken together, these experiments indicate a time-associated defect in net levels of muscle myosin for all mutants.

**Table 1.**
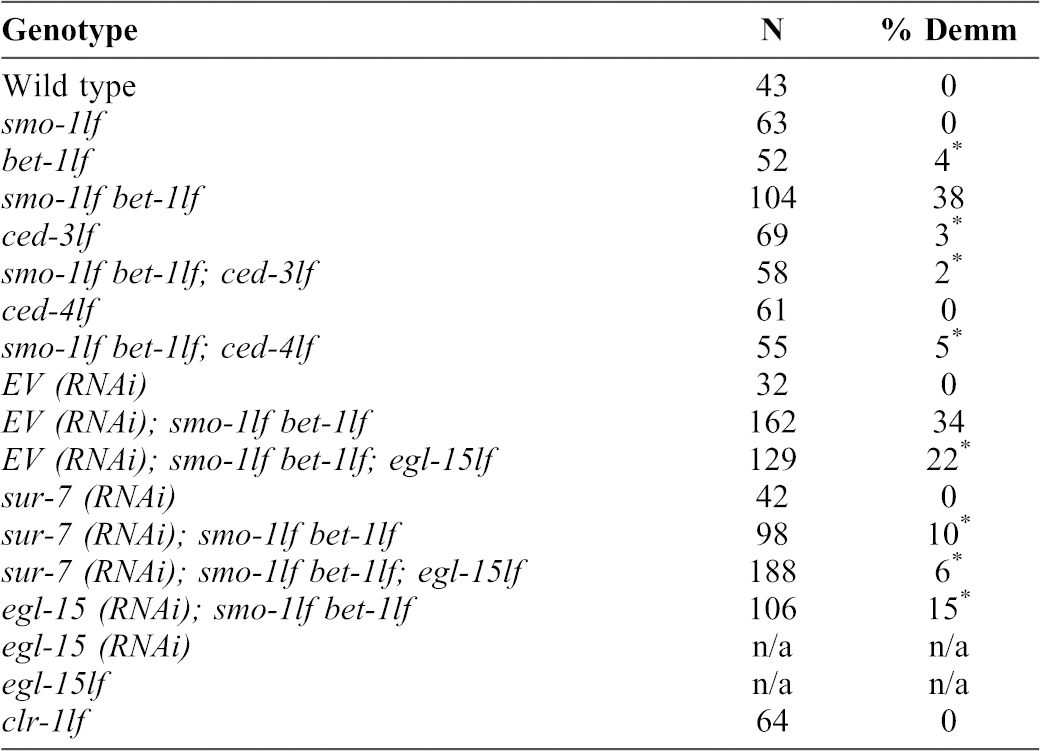
Immunostaining analysis of muscle myosin levels at day four adulthood to assess the muscle myosin depletion phenotype. Not applicable (n/a) for *egl-15* mutant or RNAi, since the worms are egg laying defective and eventually burst before any analysis can be conducted. Sterility avoids this problem. The ^*^ indicates a *P* value<0.01 by Chi square test of association. Demm: Depletion of muscle myosin.

We next verified whether the observations obtained by immunostainings could also be detected by Western blot analysis, hence eliminating possible artefacts due to the staining procedure. We prepared whole worm extracts from double *smo-1_lf_ bet-1_lf_* mutants and compared with extracts from wild type animals at three time points: L4, day-one adults and day-four adults. We found that muscle myosin levels are similar at L4 and day-one adults, but depleted at four-day adults (Fig. 1D). We quantified this effect relative to wild type sample and found that levels of myosin are down to 68.8% in average for *smo-1_lf_ bet-1_lf_* samples (SEM 6.5%; *n* = 4). This is consistent with our immunostaining experiments (Fig. 1B) and thus validates the occurrence of the muscle myosin depletion phenotype. We also observed degradation products from day-one adults, but only in wild type animals, we do not know the significance of this reproducible effect. Of note, we also confirmed the muscle myosin depletion phenotype using a different antibody against MYO-3 (supplementary material Fig. S2A). Moreover, we used an antibody against paramyosin (supplementary material Fig. S2B,C) and observed the same effect, *i.e.* immunofluorescence shows that ∼30% of double *smo-1_lf_ bet-1_lf_* mutants are depleted in paramyosin and Western blot analysis shows that paramyosin is down to 66% in average (SEM 2.8%; *n* = 4).

Finally, our data from immunostaining and Western blot analysis show that the muscle myosin depletion phenotype has an onset in adults, but it does not rule out that the casual defect occurs prior to adulthood, since the genetic deletions are always present. To circumvent this problem, we performed acute *smo-1(RNAi)* treatments on young *bet-1_lf_* adults followed by immunostainings against MYO-3 on day four adults. We observed that 28.1% (*n* = 34) of *bet-1_lf_*; *smo-1(RNAi)* are MYO-3 depleted compared with 0% (*n* = 32) for the control RNAi treatment (*P* = 0.001; Chi square test of association). This experiment using acute RNAi treatments against *smo-1* on young *bet-1_lf_* adults shows that the depletion of muscle myosin phenotype can occur after the establishment of muscle development and therefore provide further evidence that the depletion of muscle myosin is consistent with a defect during adulthood.

### Caspase-dependent depletion of muscle myosin

**Fig. 2.**
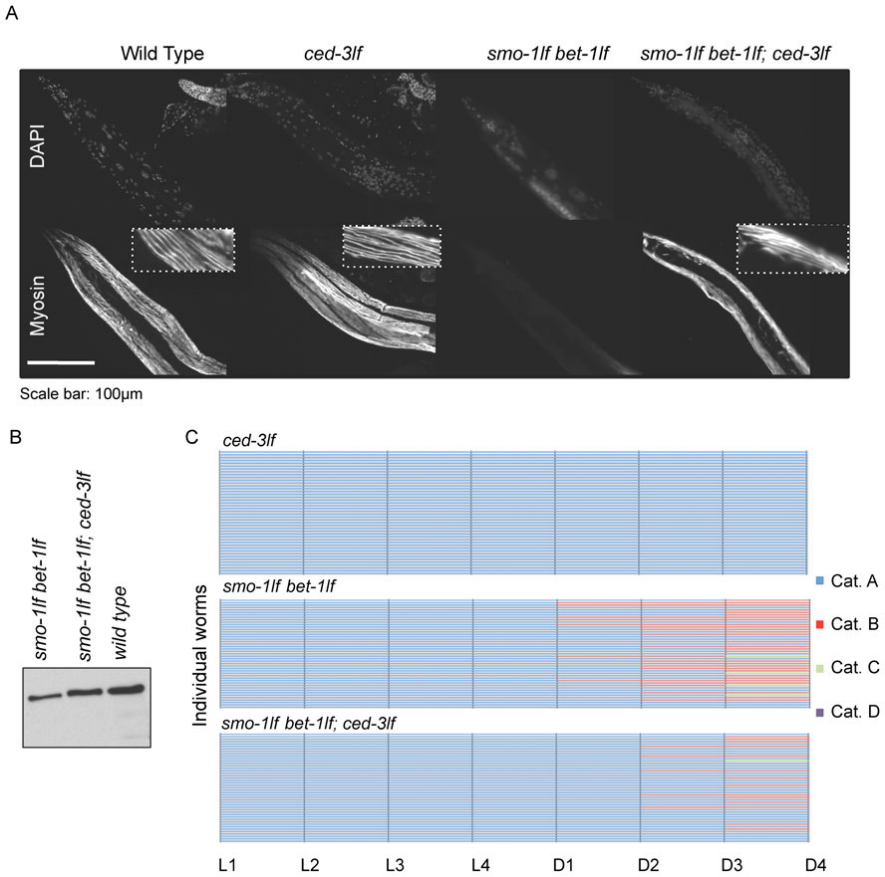
Inactivation of CED-3 in double *smo-1_lf_ bet-1_lf_* mutants prevents depletion of muscle myosin and improves locomotion. (A) Immunostaining against MYO-3 showing that the depletion of muscle myosin phenotype requires the caspase CED-3 to manifest. (B) Western blot analysis showing that muscle myosin levels are increased when compared with the double *smo-1_lf_ bet-1_lf_* mutants. See Table 1 for immunostaining results. (C) Locomotion assay performed on cloned individual animals (as in Fig. 1A) showing an improvement of locomotion when CED-3 is inactivated. Blue depicts crawling animals, red, animals requiring prodding, green, immobile animals, and purple, dead animals. L1 to L4 stands for larval stages one to four, and D1 to D4 stands for adult day one to four. Scale bar: 100 µm.

Muscle myosin levels are regulated by both synthesis and proteolysis. Since the phenotype described herein has an onset in adulthood and that the bulk of muscle myosin is synthesised prior to adulthood, we hypothesised that the depletion of muscle myosin is more consistent with excessive degradation of muscle myosin. There are four proteolysis systems described for mammalian muscles: the proteasome (Mitch and Goldberg, 1996), the lysosome (Sandri, 2013), calpains (Sorimachi and Ono, 2012) and caspases (Du et al., 2004). The first three have been shown to function in *C. elegans* (Etheridge et al., 2012). We reasoned that the proteasome and lysosome systems are unlikely to be the primary system acting on muscle myosin because of their inefficiency at directly targeting myofibrils components, such as muscle myosin (Du et al., 2004). Calpains are activated by disruption of the integrin attachment complex (Etheridge et al., 2012), which produces a very different muscle phenotype than the muscle myosin depletion phenotype. We therefore investigated whether a caspase-mediated system could be erroneously activated in *smo-1_lf_ bet-1_lf_* double mutants. To test this, we blocked the caspase cascade in *smo-1_lf_ bet-1_lf_* mutants using *ced-3* or *ced-4* loss of function mutants. CED-3 (the downstream caspase) and CED-4 (the apoptotic protease-activating factor 1) are required for most apoptosis events occurring in *C. elegans* (Ellis and Horvitz, 1986; Miura et al., 1993). The triple *smo-1_lf_ bet-1_lf_*; *ced-3_lf_* or *smo-1_lf_ bet-1_lf_*; *ced-4_lf_* mutants were analysed by immunostaining against muscle myosin. We observed that most of these caspase-defective triple mutants maintain muscle myosin levels at day four adult (CED-3: 98% and CED-4: 95%; Fig. 2A; Table 1). We verified this result using Western blots against MYO-3 and confirmed that muscle myosin levels are in average higher in absence of SMO-1, BET-1 and CED-3 than in absence of SMO-1 and BET-1 (127%, SEM 3%, *n* = 4: Fig. 2B), albeit at levels remaining below the wild type levels (Fig. 2B). Similar results were obtained using the anti-paramyosin antibody (131%, SEM 8.6%, *n* = 3; supplementary material Fig. S2D). Furthermore, we performed the locomotion assay on the triple *smo-1_lf_ bet-1_lf_*; *ced-3_lf_* mutants and observe a significant increase in the proportion of animals retaining locomotion for the triple *smo-1_lf_ bet-1_lf_*; *ced-3_lf_* mutants, when compared with the double *smo-1lf bet-1lf* mutants (from 67%, *n* = 45 to 27%, *n* = 45 at *P* = 0.0003; Fisher's exact Test) (Fig. 2C), indicating that inactivation of the caspase system causes a delay rather than a rescue of the loss of locomotion phenotype. We also present the delay effect as animated box plots for each locomotion category and strain (supplementary material Movie 2). Taken together, these results provide strong evidence that a caspase system is functional to maintain muscle homeostasis in *C. elegans*.

### The muscle myosin depletion phenotype is MEK-dependent

**Fig. 3.**
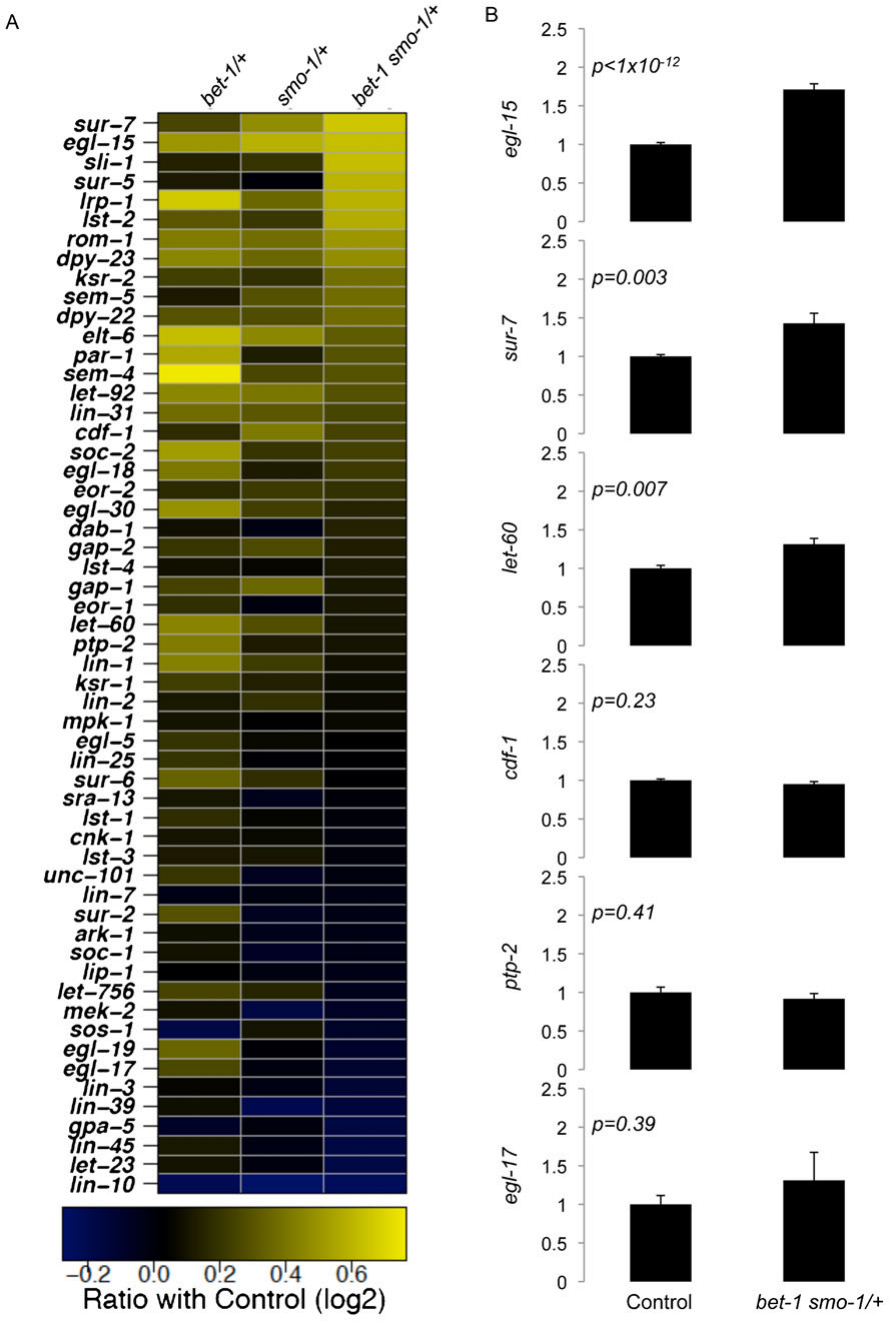
Up-regulation of *egl-15* and *sur-7* in *smo-1_lf_ bet-1_lf_* mutants. (A) Transcription profiles obtained from microarrays of *smo-1_lf_ /+*, *bet-1_lf_ /+* and *smo-1_lf_ bet-1_lf_ /+* indicate up-regulation of *egl-15* and *sur-7*. The balance strain (*+/hT2*) is used as a reference. Displayed as a colour coded matrix are the results from 56 genes known to act within the LET-60 signalling pathway. Black indicates no change, blue and yellow indicate down- and up-regulation in log2 scale, respectively. The raw data and calculated p-values are available in supplementary material Table S3. Microarray analysis for differential expression was performed using the LIMMA (Linear Models for MicroArray (Smyth, 2004)) package with Bioconductor in R. (B) Quantitative RT-PCR confirming up-regulation of *egl-15* and *sur-7* in *smo-1_lf_ bet-1_lf_* mutants. At least three independent samples were used. P values calculated using the Student T test and error bars are +/− SEM.

Both sumoylation and BET-1 are important regulators of transcription. We therefore postulated that changes in their transcription profiles could provide insights into the muscle myosin depletion phenotype. However, we met a technical problem; we could not extract sufficient materials from single or double *smo-1_lf_ bet-1_lf_* homozygous escapers. To palliate to this issue, we instead used heterozygotes (see materials and methods). As anticipated, transcription is not strikingly affected in these heterozygous animals. However, focusing our analysis on known components of the LET-60 signalling pathway, we found two possibly up-regulated positive regulators of the pathway: *egl-15*, (the FGF receptor; (DeVore et al., 1995)) and *sur-7* (a cation transporter (Yoder et al., 2004)) (Fig. 3A). To verify these results, we performed quantitative RT-PCR on independent biological samples (in the double *smo-1_lf_ bet-1_lf_* heterozygous background) and tested the levels of expression of six genes, including the *egl-15* and *sur-7* candidates (Fig. 3B). We found that both *egl-15* and *sur-7* are up-regulated significantly by about 2- and 1.5-fold, respectively (Fig. 3B), *egl-15* showing a very strong p-value (Fig. 3B). We also detected a slight up-regulation (∼1.3-fold) for *let-60* (Fig. 3B). In contrast, *cdf-1*, *ptp-2* and *egl-17* are not significantly affected (Fig. 3B). The up-regulation of *egl-15* is of interest since the FGF receptor can activate the LET-60/MEK signalling pathway and when hyperactivated increase muscle cells proteolysis as measured using a reporter assay (Kokel et al., 1998; Sundaram, 2006; Szewczyk and Jacobson, 2003). On the other hand, SUR-7 has not been linked to proteolysis in muscle cells, yet it acts as a positive regulator of LET-60/MEK signalling by regulating levels of cytoplasmic zinc ions through sequestration in the endoplasmic reticulum (Yoder et al., 2004). It is also known that elevated levels of zinc ions increase phosphorylation of the scaffold protein KSR, preventing its association with RAF and MEK and instead favouring an inhibitory association with 14-3-3 (Müller et al., 2001). Thus, up-regulation of both EGL-15 and SUR-7 are consistent with an increase in LET-60/MEK-mediated signalling.

**Fig. 4.**
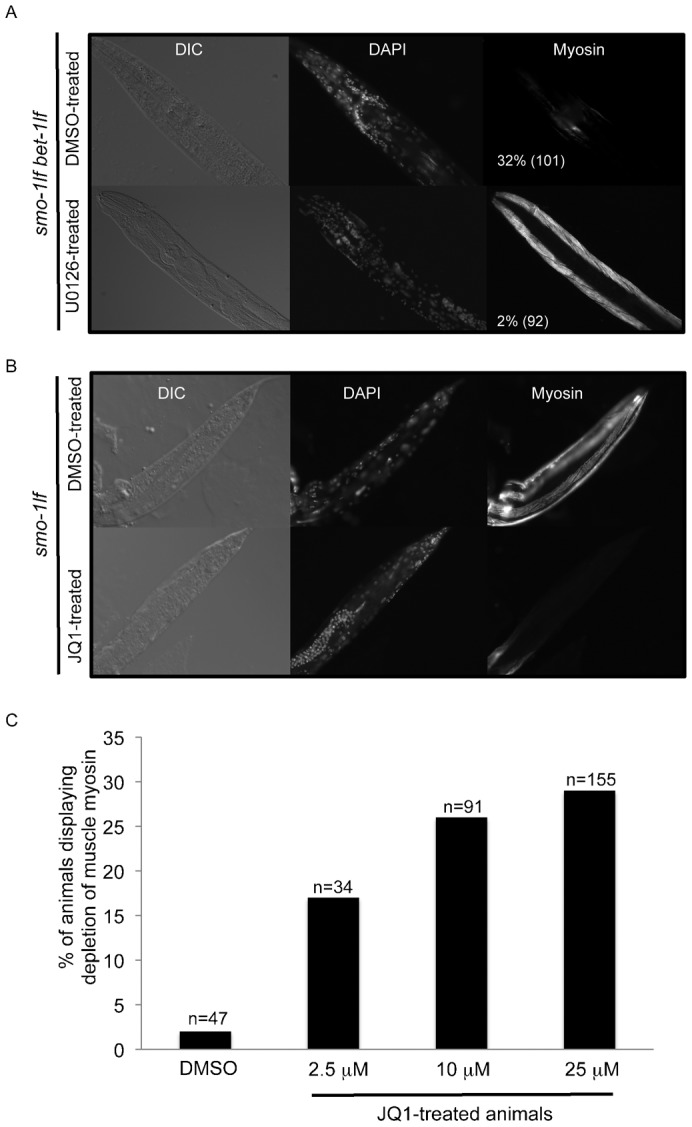
Immunofluorescence against MYO-3 on U0126-treated *smo-1_lf_ bet-1_lf_* mutants or JQ1-treated *smo-1_lf_* mutants. (A) U0126-treated *smo-1_lf_ bet-1_lf_* mutants do not display the depletion of muscle myosin phenotype. Percentages refer to animals displaying the depletion of muscle myosin phenotype with the number of animals analysed indicated. (B) Photograph of JQ1-treated *smo-1_lf_* mutant that mimics the depletion of muscle myosin phenotype. (C) The *smo-1_lf_* animals were exposed to either the control DMSO or JQ1 at the indicated concentrations. At least 50 animals were analysed for each condition. JQ1-treated wild type worms do not display the depletion of muscle myosin phenotype (not shown).

These results from the expression profile data suggested the possibility that the absence of BET-1 and SMO-1 could lead to hyperactivation of the EGL-15/LET-60/LIN-45/MEK-2 signalling pathway that in turn could initiate muscle myosin depletion. We dampened the LET-60 signalling pathway using the MEK inhibitor U0126 (Morgan et al., 2010) and measured the effect on muscle myosin levels at day four adult. Remarkably, we found that U0126-treated *smo-1_lf_ bet-1_lf_* double mutants can maintain muscle myosin levels. 32% of DMSO-treated *smo-1_lf_ bet-1_lf_* animals displayed depletion of muscle myosin compared with 2% of U0126-treated animals (Fig. 4A). Importantly, this experiment indicates that the conserved FGF receptor/RAS/RAF/MEK signalling pathway is required for the muscle myosin depletion phenotype to manifest. It is also consistent with other studies linking hyperactivation of the LET-60 signalling pathway with protein degradation in muscles (Szewczyk and Jacobson, 2003; Szewczyk et al., 2007; Szewczyk et al., 2002).

Since we found that *egl-15* and *sur-7* are overexpressed in double mutants (Fig. 3A), we next addressed specifically whether these could play an important role in the muscle myosin depletion phenotype. To this end, we depleted *smo-1_lf_ bet-1_lf_* double mutants of *egl-15* by either performing RNAi or using a reduced function allele; we found that the penetrance of the myosin depletion phenotype is decreased by 56%, from 34% to 15%, (*P* = 0.001; Table 1) and by 35%, from 34% to 22%, (*P* = 0.042; Table 1), respectively. We also tested *sur-7 (RNAi)* and observed a decrease in the penetrance by 70%, from 34% to 11% (*P*<0.001; Table 1). Thus, the overexpression of EGL-15 and SUR-7, the rescue experiments by the MEK inhibitor, and depletion of either EGL-15 or SUR-7 taken together provide strong evidence that the EGL-15/LET-60/MEK signalling pathway is required for the muscle myosin depletion phenotype to fully manifest.

### Hypodermal depletion of *egl-15* or *sur-7* rescues the muscle myosin phenotype

It has been previously shown that the EGL-15/LET-60/LIN-45/MEK-2 signalling cascade, in addition to producing the muscle cell proteolysis defect, can also cause a Clr phenotype (Huang and Stern, 2004; Kokel et al., 1998). Of note, *clr-1* mutants do not display the muscle myosin depletion phenotype (Table 1) and are morphologically different from the double *smo-1lf bet-1lf* mutants (supplementary material Fig. S3). The exact relationship between the muscle proteolysis defect and the Clr phenotype remains unclear. However, the anatomical locus of activity for the EGL-15 signalling cascade, to produce the Clr phenotype, is the hypoderm (Huang and Stern, 2004) rather than the muscles itself. With this in mind, we sought to identify the tissue in which EGL-15 and SUR-7 are required to produce muscle myosin depletion. To this end, we performed hypodermal and body wall muscle specific RNAi against *egl-15* and *sur-7*. This established tissue specific RNAi system takes advantage of an RNAi insensitive strain lacking RDE-1, in which tissue-specific re-expression of RDE-1 reactivates RNAi sensitivity in the targeted tissue (Qadota et al., 2007). We assessed whether knocking down *egl-15* or *sur-7* in either the hypoderm or the body wall muscles could rescue the muscle myosin depletion phenotype. We found that only hypodermal RNAi, of either *egl-15* or *sur-7*, can do so. Depleting EGL-15 or SUR-7 reduces the penetrance by 47% and 50%, from 32% down to 17% (*P* = 0.016) and 16% (*P* = 0011), respectively (Table 2). No rescuing effect could be detected by targeting either *egl-15* or *sur-7* in body wall muscles. Since we cannot rule out discrepancies in RNAi efficiency, we cannot rule out the possibility of a muscle activity (Table 2). Despite this caveat, these data show that hypodermal EGL-15 (and SUR-7) signalling is implicated in the depletion of muscle myosin phenotype.

**Table 2.**
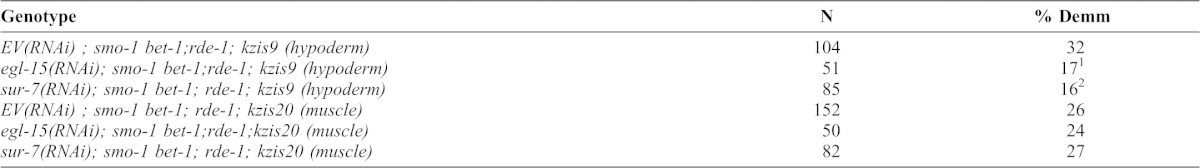
EGL-15 and SUR-7 are active in the hypoderm. The depletion of muscle myosin (Demm) phenotype was assessed using immunostaining against muscle myosin. Significant p values of 0.011^1^ and 0.016^2^ by chi square test of association.

### JQ1-treated SUMO mutants display the muscle myosin depletion phenotype

So far we have shown that BET-1 acts together with the sumoylation pathway to prevent muscle myosin depletion in adults. We next wanted to address whether the recognition of acetyl-lysines is important in the depletion of muscle phenotype. We blocked reading of acetyl-lysines using a small molecule compound inhibitor of BET proteins, JQ1 (Dawson et al., 2011; Delmore et al., 2011; Filippakopoulos et al., 2010; Nicodeme et al., 2010; Zuber et al., 2011). If recognition of acetyl-lysines is involved, we should detect the muscle myosin depletion phenotype when *smo-1_lf_* animals are treated with increasing amount of JQ1 (2.5, 10 and 25 µM). Using immunostaining, we found that JQ1-treated *smo-1_lf_* animals indeed display the muscle myosin depletion phenotype (Fig. 4B,C). JQ1 treatment of wild type animals did not cause the depletion of muscle myosin phenotype (data not shown). From this, we concluded that recognition of acetyl-lysines is important to prevent depletion of muscle myosin in adults. Since it is likely that most of these recognised acetyl-lysines are on histone tails, the data suggest that the muscle myosin depletion phenotype implicates a defect at the epigenetic level. Of note, we confirmed that JQ1 can block BET-1's association with acetyl-lysines on histones using FRAP (supplementary material Fig. S4), in accordance with another study showing that BET-1 can associate with acetylated histones (Shibata et al., 2010).

## Discussion

This study provides novel mechanistic insights into the pathways that ensure maintenance of muscle myosin levels in ageing adults and likely to influence the complex behaviour of locomotion. We present a novel muscle phenotype characterised by the depletion of adult muscle myosin. Our investigation shows a number of specific characteristics associated with this phenotype: it is caspase- and MEK-dependent, it requires hypodermal EGL-15 activity, and the muscle myosin depletion is observed only in adults and getting progressively more severe as the animals are ageing.

### Transcriptional regulation of EGL-15 and SUR-7

We have found that in absence of BET-1 and SMO-1 the FGF receptor, *egl-15*, and the cation diffusion facilitator *sur-7* are up-regulated. Since BET-1 associates with acteyl-lysines on histone tails (supplementary material Fig. S4) (Shibata et al., 2010), it is a possibility that their expression are regulated by this histone modification and therefore implicating an epigenetic mechanism. This possibility is consistent with our experiments showing that the ability to recognise acetyl-lysines is crucial to prevent muscle myosin depletion in adults (Fig. 4B,C). However, the up-regulation of both *sur-7* and *egl-15* (Fig. 3) suggests that acetyl-lysines could be interpreted as a signal for repression by BET-1, even though acetyl-lysines on histone tails are generally associated with activation of transcription. An alternative explanation for this repression effect is that BET-1 could be required to maintain the expression of a repressor that in turn acts on *sur-7* and *egl-15*. Further work will be needed to distinguish between these mechanisms.

### Non-cell autonomous EGL-15 activity

It is intriguing that the depletion of muscle myosin phenotype is apparent only in adults (in non-dividing cells), suggesting an important role for BET-1 and SMO-1 in muscle myosin homeostasis. Furthermore, we show that this phenotype is likely to involve a non-cell-autonomous mechanism. Interestingly, previous mosaic analysis on the Clear phenotype caused by hyperactivation of LET-60 signalling revealed that the anatomical locus of activity for the EGL-15 signalling cascade is hypodermal (Huang and Stern, 2004). Similarly, muscle myosin depletion is influenced by hypodermal EGL-15 activity (Table 2). Even though it is not obvious how hyperactivation of the EGL-15 signalling in the hypoderm can lead to muscle myosin depletion, there is a physical association between the muscles and the hypoderm. A recent report has shown that calpains mediate integrin attachment complex maintenance of adult muscles in *C. elegans* (Etheridge et al., 2012). Integrin attachment complexes fulfil multiple functions in muscles (Moerman and Williams, 2006), one of which is to anchor body wall muscles to the basement membrane. Since hypodermal cells are also linked to the basement membrane (Moerman and Williams, 2006) this physical association could mediate signalling events between muscles and hypodermis. Hence, hyperactivation of the EGL-15/LET-60/MEK signalling pathway in the hypoderm could produce a defect in signalling events between muscle and hypoderm, leading to the muscle myosin depletion phenotype.

### Premature loss of locomotion and depletion of muscle myosin

We have found that loss of locomotion occurs prematurely in single and especially in double *smo-1_lf_ bet-1_lf_* mutants. Loss of locomotion can be observed before the depletion of muscle myosin. This sequence of events strongly suggests that another function impacting on locomotion is impaired in these mutants. Locomotion is a complex behaviour involving muscles, neurons and muscle attachments to the cuticle *via* the hypoderm. We have shown that a defect in signalling occurs in the hypoderm and that muscle cells are depleted in muscle myosin. It remains to be investigated whether neurons are affected, since BET-1 has been shown to act in stabilising neuronal cell fate (Shibata et al., 2010). It is also a possibility that the loss of locomotion is the primary defect, leading to the depletion of muscle myosin. There is however a discrepancy between the percentage of animals losing locomotion and the percentage of animals depleted in muscle myosin; there are at least twice as many animals losing locomotion that there are animals depleted in muscle myosin. Further, inactivation of CED-3 in *smo-1_lf_ bet-1_lf_* mutants allowed maintenance of muscle myosin levels. However, we observe the same discrepancy aforementioned between loss of locomotion at day four and depletion of muscle myosin. Thus, the depletion of muscle myosin, at day four adult, appears unlikely to be induced by loss of locomotion. Unless, a number of animals have been in an immobilised state longer than others prior to analysis and that those particular animals are depleted in muscle myosin.

Finally, the muscle myosin depletion phenotype that we described produces an effect resembling muscle atrophy. However, it is unclear whether this phenotype is actually a premature manifestation of sarcopenia, the loss of muscle mass due to ageing, which occurs naturally in *C. elegans* (Herndon et al., 2002) or whether it is a muscular pathology. Taken together, our study has identified *bet-1* and *smo-1* as important players in the maintenance of adult muscle myosin levels through a caspase- and MEK-dependent mechanism, which could be relevant to muscle ageing and/or a muscle pathology.

## Supplementary Material

Supplementary Material
