# Supplementary material for: Maintenance of muscle myosin levels in adult C. elegans requires both the double bromodomain protein BET-1 and sumoylation
Source: Biol Open. 2013 Oct 31;2(12):1354–63. doi: 10.1242/bio.20136007 (PMC3863420; doi:10.1242/bio.20136007)
Supplement: Supplementary Material [file supp_2_12_1354__index.html]

Maintenance of muscle myosin levels in adult C. elegans requires both the double bromodomain protein BET-1 and sumoylation — Supplementary Material 

# Maintenance of muscle myosin levels in adult *C. elegans* requires both the double bromodomain protein BET-1 and sumoylation

## bio.20136007 Supplementary Material

**Files in this Data Supplement:**

- Supplementary Material - Kate Fisher et al. doi: 10.1242/bio.20136007
- Movie 1 - **Animated box plots showing the life histories of the wild type and mutant worm populations at each day of the 21-day locomotion assay.** Each frame represents one day of 21 days of analysis. The life history is presented in terms of days spent in any of the four categories from the locomotion assay depicted in Fig. 1A. Each box plot describes the distribution of the days spent in that category by the indicated worm populations. The central bar in bold represents the median of days spent in a particular category. The upper and lower limits of the box represent the 3rd quartile and 1st quartile respectively. The ends of the whiskers above and below denote the highest and lowest values in the data, although anything beyond 1.5-fold of inter-quartile range above the third quartile or below the first quartile is defined as an outlier and plotted as circles. For example, by selecting day 10 of the assay, we see that the wild type population of worms has spent all 10 days in category A and none in any of the other categories. As for the double *smo-1lf bet-1lf* mutant, we see that the majority of the population has only spent 5–7 days of their lives in category A. Of those that progress to either category B or C, they have spent a median of 3 days in each. An outlier of the population has progressed to category D and spent 1 day like this, although the majority have not progressed so far yet.
- Movie 2 - **Animated box plots (as in supplementary material Movie 1) showing the life histories of the wild type and mutant worm populations at each day of the 21-day locomotion assay.** Each frame represents one day of 21 days of analysis. The life history is presented in terms of days spent in any of the four categories from the locomotion assay depicted in Fig. 2C. Each box plot describes the distribution of the days spent in that category by the indicated worm populations. The central bar in bold represents the median of days spent in a particular category. The upper and lower limits of the box represent the 3rd quartile and 1st quartile respectively. The ends of the whiskers above and below denote the highest and lowest values in the data, although anything beyond 1.5-fold of inter-quartile range above the third quartile or below the first quartile is defined as an outlier and plotted as circles.
- Table S1
- Table S2
- Table S3
- Table S4
