# Supplementary material for: Maintenance of muscle myosin levels in adult C. elegans requires both the double bromodomain protein BET-1 and sumoylation
Source: Biol Open. 2013 Oct 31;2(12):1354–63. doi: 10.1242/bio.20136007 (PMC3863420; doi:10.1242/bio.20136007)
Supplement: Supplementary Material [file supp_bio.20136007_bio.20136007-s1.pdf]

Supplementary Material

Kate Fisher et al. doi: 10.1242/bio.20136007

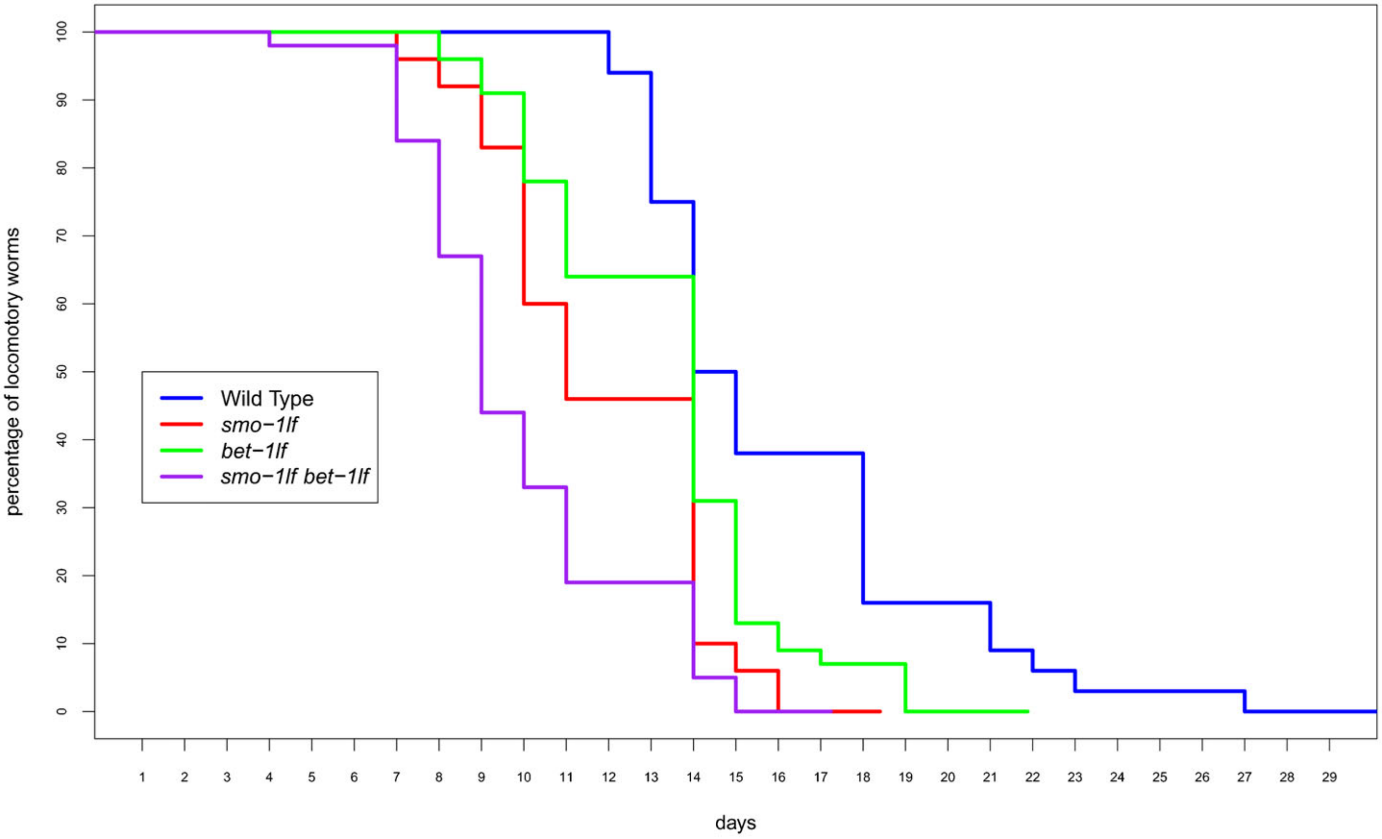

Fig. S1. Locomotion assay as in Fig. 1 but the categories B and C were pooled. The data are displayed by a cumulative plot of locomotory versus locomotory impaired showing that the double *smo-1*$_{lf}$ *bet-1*$_{lf}$ mutants are more affected than their respective single mutants. All mutants are affected when compared with the wild type.

Biology Open

Biology Open

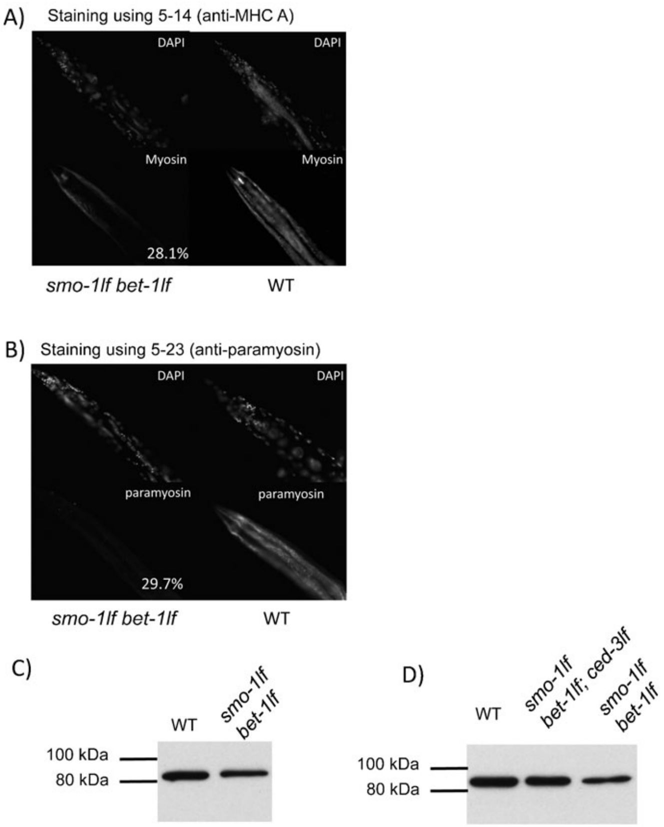

Fig. S2. Different antibodies against muscle myosin or paramyosin show results consistent with Fig. 1. (A) Immunostaining using a different antibody against MYO-3 (5–14). (B) Immunostaining using an antibody against paramyosin (5–23). (C,D) Western blots using an antibody against paramyosin (5–23). (C) Western blots using 5–23 (antiparamyosin) were performed in quadriplicates and show that paramyosin is depleted in double *smo-1lf bet-1lf* mutants as found with muscle myosin. Quantification relative to Wild Type: 66%, SEM +/− 2.8%. (D) Western blots using 5–23 (antiparamyosin) were performed in triplicates and show that paramyosin is depleted in double *smo-1lf bet-1lf* mutants as found with muscle myosin and expression rescuedin the triple *smo-1lf bet-1lf; ced-3lf*. Quantification of triple relative to double mutant: 131%, SEM +/− 8.6%.

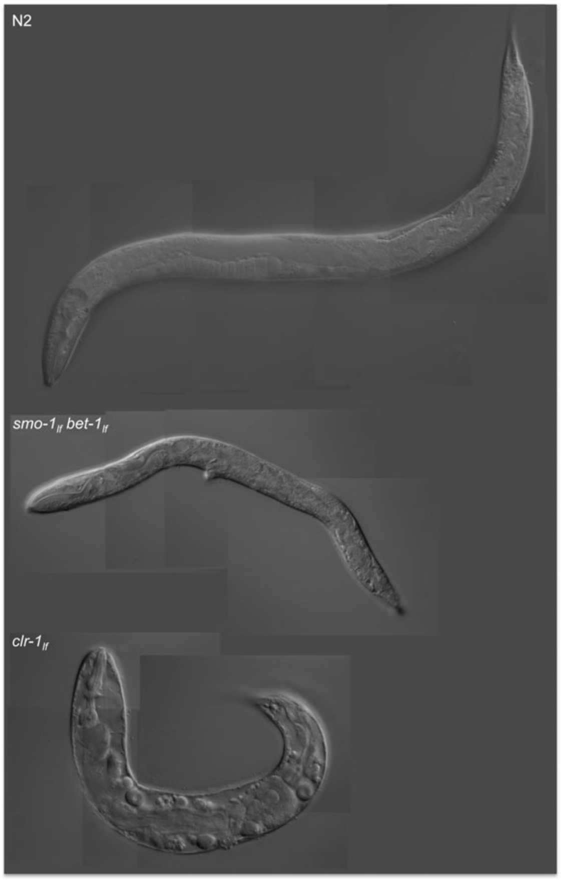

Fig. S3. DIC photographs showing the morphological appearance of the double *smo-1$_{lf}$ bet-1$_{lf}$* mutants compared with wild type and the *clr-1$_{lf}$* mutant.

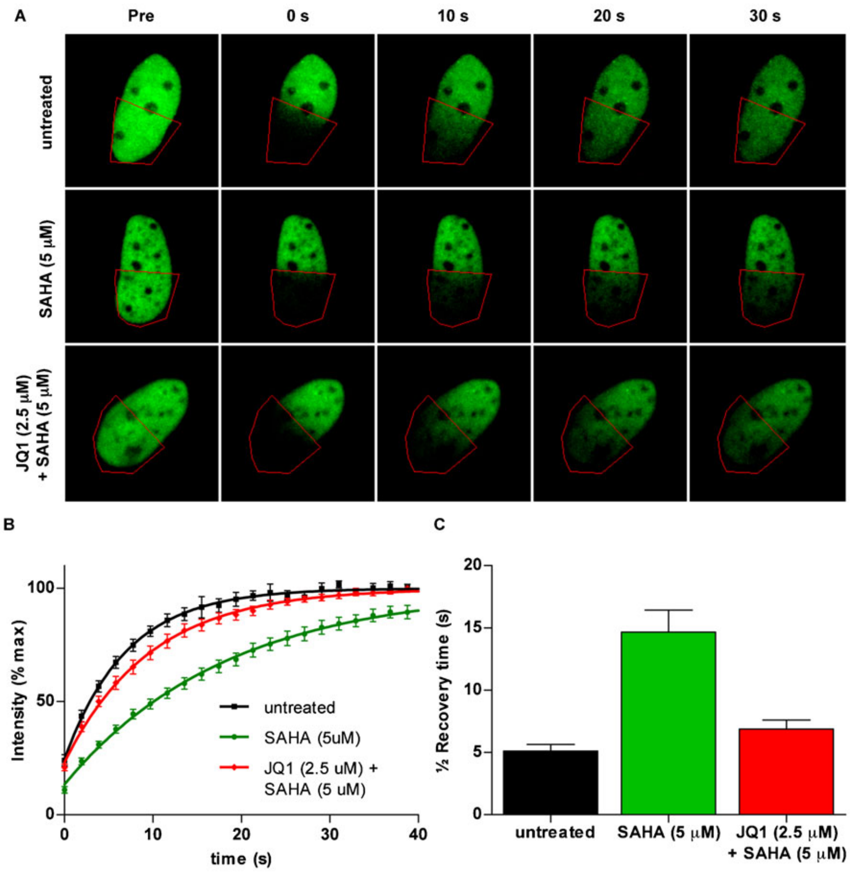

Fig. S4. BET-1 can be displaced from chromatin by the bromodomain inhibitor JQ1. (A) Photographs of different time courses of representative cells showing FRAP (Fluorescence Recovery After Photobleaching) for BET-1::GFP. Untreated control human U2OS cells recover rapidly during FRAP, but the recovery rate for SAHA-treated cells is reduced. The HDAC inhibitor SAHA is used to increase the levels of global acetylation (by preventing deacetylation), which stabilises BET-1::GFP association with acetylated chromatin. The principle of the assay is based on the capacity of BET-1::GFP to diffuse freely if not stably associated with histones. When cells are treated with SAHA, BET-1::GFP's association with acetyl-lysine on histone tails is stabilised and the half time for recovery is increased. When JQ1 is added, we found a faster recovery rate, indicating that JQ1 destabilises BET-1::GFP's association with acetylated histones. (B) Time course of fluorescence intensity after photobleaching, shown are the mean of at least 10 cells per treatment. (C) Mean time for half-maximal recovery of each treatment group calculated from the time courses of individual cells. These data are consistent with structural studies showing that an acetyl-lysine molecule is recognized by a central hydrophobic cavity and anchored by a hydrogen bond using a conserved asparagine residue present in bromodomains (Owen et al., 2000). This asparagine residue is present in BET-1 and our results are therefore consistent with these structural predictions.

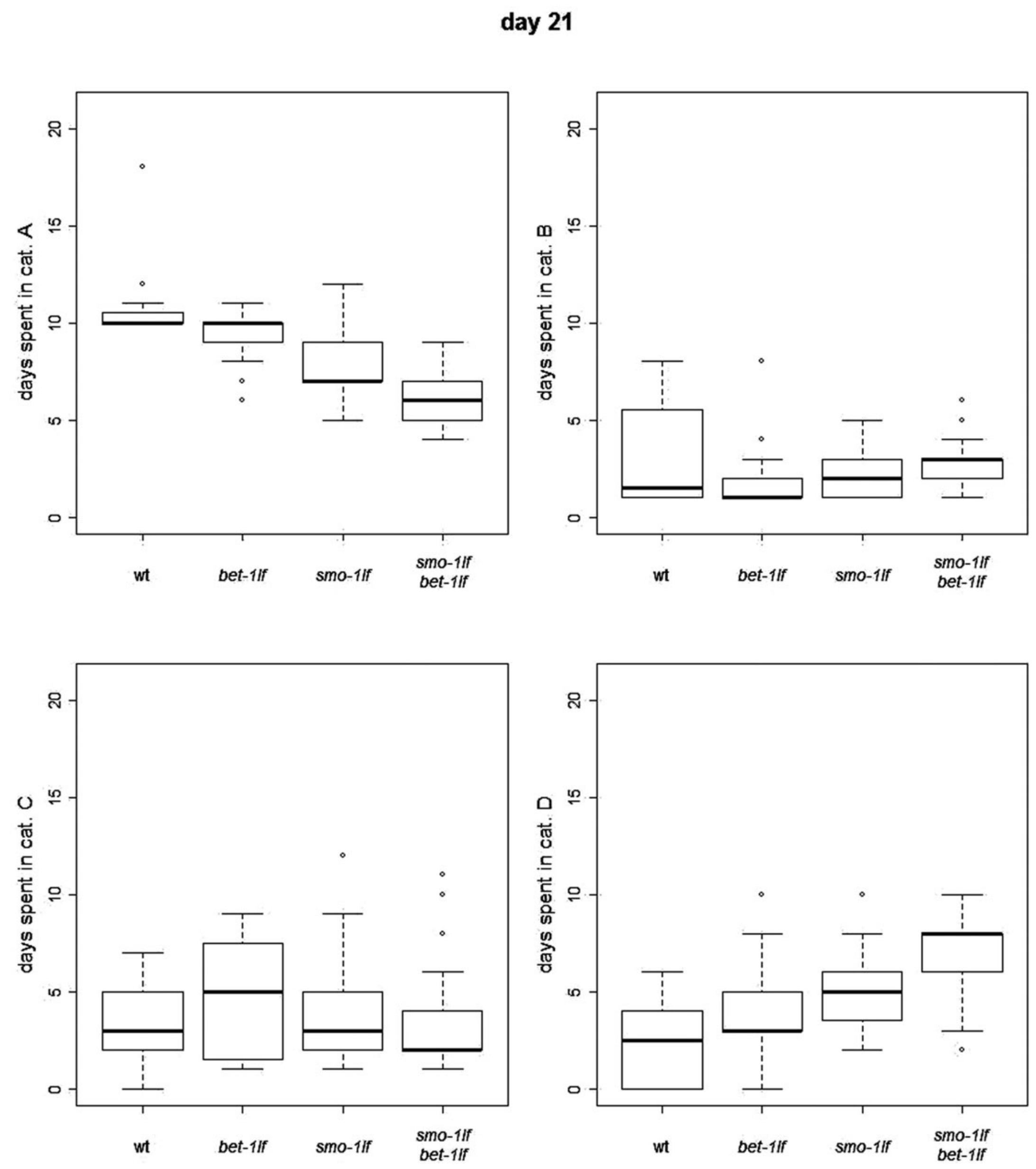

Movie 1. Animated box plots showing the life histories of the wild type and mutant worm populations at each day of the 21-day locomotion assay. Each frame represents one day of 21 days of analysis. The life history is presented in terms of days spent in any of the four categories from the locomotion assay depicted in Fig. 1A. Each box plot describes the distribution of the days spent in that category by the indicated worm populations. The central bar in bold represents the median of days spent in a particular category. The upper and lower limits of the box represent the 3rd quartile and 1st quartile respectively. The ends of the whiskers above and below denote the highest and lowest values in the data, although anything beyond 1.5-fold of inter-quartile range above the third quartile or below the first quartile is defined as an outlier and plotted as circles. For example, by selecting day 10 of the assay, we see that the wild type population of worms has spent all 10 days in category A and none in any of the other categories. As for the double $smo\text{-}1_{lf}$ $bet\text{-}1_{lf}$ mutant, we see that the majority of the population has only spent 5–7 days of their lives in category A. Of those that progress to either category B or C, they have spent a median of 3 days in each. An outlier of the population has progressed to category D and spent 1 day like this, although the majority have not progressed so far yet.

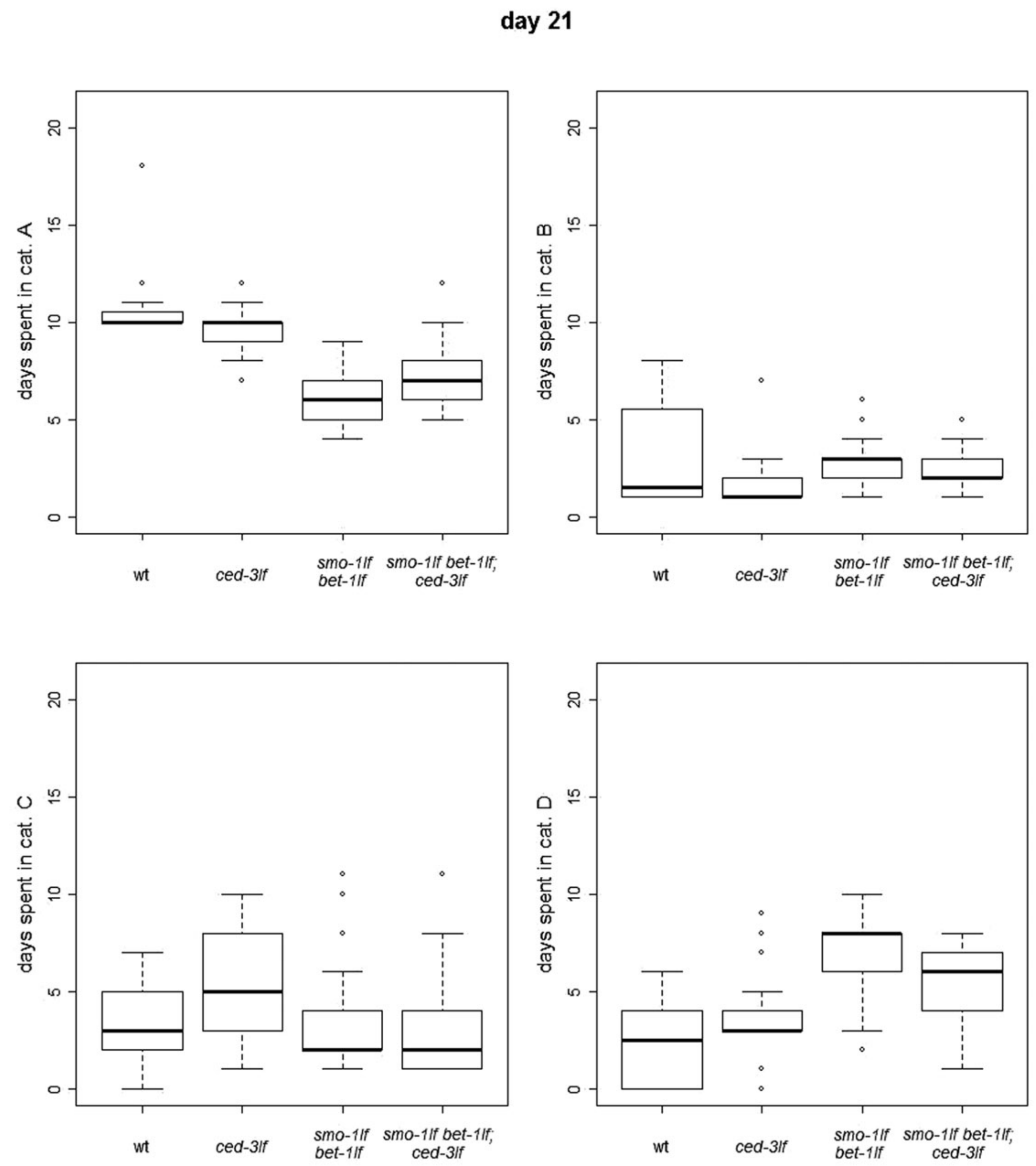

Movie 2. Animated box plots (as in supplementary material Movie 1) showing the life histories of the wild type and mutant worm populations at each day of the 21-day locomotion assay. Each frame represents one day of 21 days of analysis. The life history is presented in terms of days spent in any of the four categories from the locomotion assay depicted in Fig. 2C. Each box plot describes the distribution of the days spent in that category by the indicated worm populations. The central bar in bold represents the median of days spent in a particular category. The upper and lower limits of the box represent the 3rd quartile and 1st quartile respectively. The ends of the whiskers above and below denote the highest and lowest values in the data, although anything beyond 1.5-fold of inter-quartile range above the third quartile or below the first quartile is defined as an outlier and plotted as circles.
